# Supplementary material for: HIVprotI: an integrated web based platform for prediction and design of HIV proteins inhibitors
Source: J Cheminform. 2018 Mar 9;10:12. doi: 10.1186/s13321-018-0266-y (PMC5845081; doi:10.1186/s13321-018-0266-y)
Supplement: Supplementary file 2 — Additional file 2. Source code of HIVProtI web server. [file 13321_2018_266_MOESM2_ESM.zip › HIVprotI_Source-code/hivprotI/weka-3-6-6/doc/weka/core/BinarySparseInstance.html]

BinarySparseInstance


---


|  |  |  |
| --- | --- | --- |
| **Overview** \| **Package** \| **Class** \| **Tree** \| **Deprecated** \| **Index** \| **Help** \| **Weka's home** | |  |
| **PREV CLASS**   **NEXT CLASS** | **FRAMES**    **NO FRAMES**     **All Classes** |  |
| SUMMARY: NESTED \| FIELD \| CONSTR \| METHOD | DETAIL: FIELD \| CONSTR \| METHOD |  |


---


## weka.core Class BinarySparseInstance

```
java.lang.Object
  weka.core.Instance
      weka.core.SparseInstance
          weka.core.BinarySparseInstance
```

**All Implemented Interfaces:**: java.io.Serializable, Copyable, RevisionHandler

---

``` public class BinarySparseInstance extends SparseInstance ```

Class for storing a binary-data-only instance as a sparse vector. A
sparse instance only requires storage for those attribute values
that are non-zero. Since the objective is to reduce storage
requirements for datasets with large numbers of default values,
this also includes nominal attributes -- the first nominal value
(i.e. that which has index 0) will not require explicit storage, so
rearrange your nominal attribute value orderings if
necessary. Missing values are not supported, and will be treated as
1 (true).

**Version:**
:   $Revision: 1.13 $

**See Also:**: Serialized Form

---

**Constructor Summary**

`BinarySparseInstance(double weight,
double[] attValues)`
  
          Constructor that generates a sparse instance from the given
parameters.

`BinarySparseInstance(double weight,
int[] indices,
int maxNumValues)`
  
          Constructor that inititalizes instance variable with given
values.

`BinarySparseInstance(Instance instance)`
  
          Constructor that generates a sparse instance from the given
instance.

`BinarySparseInstance(int numAttributes)`
  
          Constructor of an instance that sets weight to one, all values to
1, and the reference to the dataset to null.

`BinarySparseInstance(SparseInstance instance)`
  
          Constructor that copies the info from the given instance.


| **Method Summary** | |
| --- | --- |
| `java.lang.Object` | `copy()`             Produces a shallow copy of this instance. |
| `java.lang.String` | `getRevision()`             Returns the revision string. |
| `static void` | `main(java.lang.String[] options)`             Main method for testing this class. |
| `Instance` | `mergeInstance(Instance inst)`             Merges this instance with the given instance and returns the result. |
| `void` | `replaceMissingValues(double[] array)`             Does nothing, since we don't support missing values. |
| `void` | `setValue(int attIndex, double value)`             Sets a specific value in the instance to the given value (internal floating-point format). |
| `void` | `setValueSparse(int indexOfIndex, double value)`             Sets a specific value in the instance to the given value (internal floating-point format). |
| `double[]` | `toDoubleArray()`             Returns the values of each attribute as an array of doubles. |
| `java.lang.String` | `toString()`             Returns the description of one instance in sparse format. |
| `double` | `value(int attIndex)`             Returns an instance's attribute value in internal format. |
| `double` | `valueSparse(int indexOfIndex)`             Returns an instance's attribute value in internal format. |

**Methods inherited from class weka.core.SparseInstance**

`attributeSparse, index, isMissing, locateIndex, numAttributes, numValues`

**Methods inherited from class weka.core.Instance**

`attribute, classAttribute, classIndex, classIsMissing, classValue, dataset, deleteAttributeAt, enumerateAttributes, equalHeaders, hasMissingValue, insertAttributeAt, isMissing, isMissingSparse, isMissingValue, missingValue, numClasses, relationalValue, relationalValue, setClassMissing, setClassValue, setClassValue, setDataset, setMissing, setMissing, setValue, setValue, setValue, setWeight, stringValue, stringValue, toString, toString, value, weight`

**Methods inherited from class java.lang.Object**

`equals, getClass, hashCode, notify, notifyAll, wait, wait, wait`

**Constructor Detail**

### BinarySparseInstance

```
public BinarySparseInstance(Instance instance)
```

:   Constructor that generates a sparse instance from the given
    instance. Reference to the dataset is set to null.
    (ie. the instance doesn't have access to information about the
    attribute types)

    **Parameters:**: `instance` - the instance from which the attribute values and the weight are to be copied

---


### BinarySparseInstance

```
public BinarySparseInstance(SparseInstance instance)
```

:   Constructor that copies the info from the given instance.
    Reference to the dataset is set to null.
    (ie. the instance doesn't have access to information about the
    attribute types)

    **Parameters:**: `instance` - the instance from which the attribute info is to be copied

---


### BinarySparseInstance

```
public BinarySparseInstance(double weight,
                            double[] attValues)
```

:   Constructor that generates a sparse instance from the given
    parameters. Reference to the dataset is set to null.
    (ie. the instance doesn't have access to information about the
    attribute types)

    **Parameters:**: `weight` - the instance's weight: `attValues` - a vector of attribute values

---


### BinarySparseInstance

```
public BinarySparseInstance(double weight,
                            int[] indices,
                            int maxNumValues)
```

:   Constructor that inititalizes instance variable with given
    values. Reference to the dataset is set to null. (ie. the instance
    doesn't have access to information about the attribute types)

    **Parameters:**: `weight` - the instance's weight: `indices` - the indices of the given values in the full vector: `maxNumValues` - the maximium number of values that can be stored

---


### BinarySparseInstance

```
public BinarySparseInstance(int numAttributes)
```

:   Constructor of an instance that sets weight to one, all values to
    1, and the reference to the dataset to null. (ie. the instance
    doesn't have access to information about the attribute types)

    **Parameters:**: `numAttributes` - the size of the instance


**Method Detail**

### copy

```
public java.lang.Object copy()
```

:   Produces a shallow copy of this instance. The copy doesn't have
    access to a dataset.

    :   **Specified by:**: `copy` in interface `Copyable` **Overrides:**: `copy` in class `SparseInstance`
    :   **Returns:**: the shallow copy

---


### mergeInstance

```
public Instance mergeInstance(Instance inst)
```

:   Merges this instance with the given instance and returns
    the result. Dataset is set to null.

    :   **Overrides:**: `mergeInstance` in class `SparseInstance`
    :   **Parameters:**: `inst` - the instance to be merged with this one **Returns:**: the merged instances

---


### replaceMissingValues

```
public void replaceMissingValues(double[] array)
```

:   Does nothing, since we don't support missing values.

    :   **Overrides:**: `replaceMissingValues` in class `SparseInstance`
    :   **Parameters:**: `array` - containing the means and modes

---


### setValue

```
public void setValue(int attIndex,
                     double value)
```

:   Sets a specific value in the instance to the given value
    (internal floating-point format). Performs a deep copy
    of the vector of attribute values before the value is set.

    :   **Overrides:**: `setValue` in class `SparseInstance`
    :   **Parameters:**: `attIndex` - the attribute's index: `value` - the new attribute value (If the corresponding attribute is nominal (or a string) then this is the new value's index as a double).

---


### setValueSparse

```
public void setValueSparse(int indexOfIndex,
                           double value)
```

:   Sets a specific value in the instance to the given value
    (internal floating-point format). Performs a deep copy
    of the vector of attribute values before the value is set.

    :   **Overrides:**: `setValueSparse` in class `SparseInstance`
    :   **Parameters:**: `indexOfIndex` - the index of the attribute's index: `value` - the new attribute value (If the corresponding attribute is nominal (or a string) then this is the new value's index as a double).

---


### toDoubleArray

```
public double[] toDoubleArray()
```

:   Returns the values of each attribute as an array of doubles.

    :   **Overrides:**: `toDoubleArray` in class `SparseInstance`
    :   **Returns:**: an array containing all the instance attribute values

---


### toString

```
public java.lang.String toString()
```

:   Returns the description of one instance in sparse format.
    If the instance doesn't have access to a dataset, it returns the
    internal floating-point values. Quotes string values that contain
    whitespace characters.

    :   **Overrides:**: `toString` in class `SparseInstance`
    :   **Returns:**: the instance's description as a string

---


### value

```
public double value(int attIndex)
```

:   Returns an instance's attribute value in internal format.

    :   **Overrides:**: `value` in class `SparseInstance`
    :   **Parameters:**: `attIndex` - the attribute's index **Returns:**: the specified value as a double (If the corresponding attribute is nominal (or a string) then it returns the value's index as a double).

---


### valueSparse

```
public final double valueSparse(int indexOfIndex)
```

:   Returns an instance's attribute value in internal format.
    Does exactly the same thing as value() if applied to an Instance.

    :   **Overrides:**: `valueSparse` in class `Instance`
    :   **Parameters:**: `indexOfIndex` - the index of the attribute's index **Returns:**: the specified value as a double (If the corresponding attribute is nominal (or a string) then it returns the value's index as a double).

---


### main

```
public static void main(java.lang.String[] options)
```

:   Main method for testing this class.

    :   **Parameters:**: `options` - the command line options - ignored

---


### getRevision

```
public java.lang.String getRevision()
```

:   Returns the revision string.

    :   **Specified by:**: `getRevision` in interface `RevisionHandler` **Overrides:**: `getRevision` in class `SparseInstance`
    :   **Returns:**: the revision


---


|  |  |  |
| --- | --- | --- |
| **Overview** \| **Package** \| **Class** \| **Tree** \| **Deprecated** \| **Index** \| **Help** \| **Weka's home** | |  |
| **PREV CLASS**   **NEXT CLASS** | **FRAMES**    **NO FRAMES**     **All Classes** |  |
| SUMMARY: NESTED \| FIELD \| CONSTR \| METHOD | DETAIL: FIELD \| CONSTR \| METHOD |  |


---
